# Dual role of CD73 as a signaling molecule and adenosine-generating enzyme in colorectal cancer progression and immune evasion

**DOI:** 10.7150/ijbs.87440

**Published:** 2024-01-01

**Authors:** Weidong Lian, Dan Jiang, Wandie Lin, Muhong Jiang, Yujie Zhang, Hui Wang, Liang Zhao

**Affiliations:** 1 Department of Pathology, Nanfang Hospital, Southern Medical University Guangzhou, China.; 2 The National Key Clinical Specialty, Department of Neurosurgery, Zhujiang Hospital, Southern Medical University Guangzhou, China.; 3 Department of Pathology & Guangdong Province Key Laboratory of Molecular Tumor Pathology, School of Basic Medical Sciences, Southern Medical University, Guangzhou, China.; 4 Department of Medical Oncology, Affiliated Tumour Hospital of Guangzhou Medical University, Guangzhou, China.

**Keywords:** colorectal cancer, CD73, tumor progression, tumor immune, immune checkpoint blockade

## Abstract

Metastasis and limited benefits of immune checkpoint blockade are two obstacles to the battle against colorectal cancer (CRC). CD73, encoded by the gene 5'-Nucleotidase Ecto (NT5E), is a major enzyme that generates extracellular adenosine. However, whether CD73 affects cancer progression and immune response in CRC remains unclear. Here, the clinical significance of CD73 was assessed in human CRC specimens using immunohistochemistry and bioinformatic analyses. We demonstrated that CD73 is elevated in CRC tissues, particularly in those with metastasis, and correlates with poor prognosis. Gain- and loss-of-function experiments demonstrate that tumor CD73 supports tumor progression and impairs the viability and effector functions of CD8^+^ T cells. Targeting CD73 on CRC cells reduces their malignant phenotypes and improves the anti-cancer response of CD8^+^ T cells in the tumor microenvironment (TME). Moreover, the combination of CD73 blockade and PD-1 inhibitors exhibited enhanced anti-cancer effects when compared to a single-agent treatment. Thus, CD73 may be a promising target in the treatment of CRC.

## Background

Colorectal cancer (CRC) is a leading cause of cancer-related deaths globally. According to Global Cancer Statistics, there were an estimated 1.9 million newly developed CRC patients worldwide in 2020, representing about one-tenth cancer cases and deaths 1. Potential of distant metastasis to liver or lung contributes to difficulties of CRC treatment. Currently, the two main treatments for CRC are surgical resection and chemotherapy 2. In recent years, emerging immune checkpoint blockade (ICB) therapies have provided new options in patients with advanced cancer 3, 4. However, ICB therapies have limited efficiency in CRC 5. Mechanistically, effective ICB therapies mainly depends on CD8^+^ T cells 6-8. Attenuating the metastatic potential of CRC cells and promoting the infiltration and effector functions of CD8^+^ T cells may be important strategies to improve clinical outcomes of CRC patients.

The adenosinergic pathway has been paid attention to as a novel immunotherapeutic target 9. Under conditions like cell death, hypoxia and starvation, adenosine triphosphate (ATP) is released by cells, especially by malignant tumor cells. The cell surface enzyme CD39 transforms extracellular ATP into adenosine monophosphate (AMP), and then AMP is dephosphorylated to adenosine by CD73. CD73, encoded by the gene NT5E, is a glycosyl-phosphatidylinositol (GPI) anchored cell surface enzyme. As large number of studies have reported, CD73 could be upregulated on both cancer cells and non-cancer cells in the TME. In addition, CD73 exerts non-enzymatic functions as a signaling molecule in oncogenic pathways 10-13. These studies revealed that CD73 may contribute to cancer progression in either an immune-independent or an immune-dependent manner. In CRC, preliminary studies reported that CD73 was upregulated and high CD73 on CRC cells promotes cell growth 14, 15. Serum samples from CRC patients presented higher CD73 activity than those from healthy controls 16. However, it is still unclear whether CD73 could facilitate the development and immunosuppression of CRC, as well as the underlying regulatory mechanism.

In this research, we utilized clinical CRC specimens, mouse CRC models, and publicly accessible bulk and single-cell RNA sequencing from human CRC-tissues to investigate expression of CD73 in CRC and its association with CRC biology and immunology. The gain- and loss-of-function of CD73 on CRC cells modulated the malignant behaviors of CRC cells and phenotypes of anti-tumor CD8^+^ T cells. Furthermore, we explored the possibility of combining ICBs with CD73 blockade. Our findings identified CD73 as a master regulator of CRC progression and immune evasion.

## Materials and methods

### Human CRC microarrays

As described in our previous study 17, clinical CRC specimens with parts of matched adjacent normal colorectal tissues were collected in the Nanfang Hospital between 2012 and 2018. These samples were then used to construct tissue microarrays. The study complied with the Declaration of Helsinki and was approved in its entirety by Southern Medical University's ethics committee.

### Cell lines and culture

Human normal colon epithelial cell line NCM460, and human CRC cell lines including SW480, SW620, RKO, HCT116, HT29, LoVo, and LS174T were purchased from the Cell Bank of the Chinese Academy of Sciences and maintained as previously described 18, and cultured in RPMI 1640 (Hyclone) supplemented with 10% fetal bovine serum (FBS) (Gibco-BRL, Invitrogen) at 37 °C with a humidity of 5% CO_2_. The MC38 cell line (C57BL/6 J murine colon carcinoma cells) was purchased from Kerafast (Boston, USA) and cultured in DMEM (Gibco) supplemented with 10% FBS (Gibco). All cell lines used were routinely tested for mycoplasma to make sure they were free of contamination.

### Plasmids and lentivirus

Plasmids containing short hairpin RNAs (shRNAs) for human CD73 silencing and CD73 cDNA for human CD73 overexpression were constructed from GeneChem Inc (Shanghai, China). Human CRC cells HCT116 and SW480 were transfected with CD73 knockdown or overexpressing plasmids, respectively, using Lipofectamine 3000 (Invitrogen) following the manufacturer's instructions.

Lentiviral particles containing short hairpin RNAs (shRNAs) for murine CD73 silencing (CD73i) were constructed from GenePharma Inc (Shanghai, China). MC38 cells were infected with control or CD73i lentivirus with addition of polybrene. Quantitative real-time PCR (qRT-PCR) analyses verified that CD73i lentivirus downregulated the level of CD73 mRNA in MC38 cells from control groups. For either human or mouse CRC cells, stable CD73 overexpression or knockdown cell lines were selected with 2 μg/mL puromycin for 7 days.

To establish OVA (ovalbumin) -expressing MC38 cells, lentivirus was produced by subcloning the cDNA of OVA into a lentiviral plasmid. MC38 cells were infected with the lentivirus and subsequently tested positive for OVA expression.

### Peripheral blood mononuclear cells (PBMCs) isolation and CD8^+^ T cells purification

Human PBMCs were isolated from healthy donors by the density gradient centrifugation using Ficoll-Paque Plus from Sigma-Aldrich (#GE17-1440-02). Human CD8^+^ T cells were isolated from whole blood from healthy donors by the magnetic bead separation using the Direct Human CD8 Isolation Kit from Stemcell (#19663). Purified CD8^+^ T cells were activated and expanded with anti-CD3/CD28 (Stemcell, Canada, #10971) and cultured in RPMI 1640 containing 10% FBS, 100 µg/mL penicillin/streptomycin and recombinant human IL-2 (PeproTech). 72 h after activation, activated CD8^+^ T cells were treated with different conditioned medium (CM).

### Cell viability assays

To determine proliferation of human CRC cells *in vitro*, 1×10^3^ cells per well were seeded into 96-well plates. Every 24 h, the CCK-8 solution (Dojindo, Japan) was added into the medium and incubated for 2 h, followed by measuring OD value at 450nm. Three independent experiments were performed.

To determine effects of tumor CD73 on T cells, four types of conditioned medium (CM) from cultured control (NC) or CD73-overexpression (OE) SW480 cells were obtained: (1) control complete medium; (2) complete medium added with 50 μM 5′-AMP; (3) complete medium added with 25 nM SCH58261; (4) complete medium added with 50 μM 5′-AMP and 25 nM SCH58261. To investigate proliferation of CD8^+^ T cells, isolated and purified human CD8^+^ T cells from healthy donors were firstly labeled with 2.5 μM CFSE (Biolegend) and activated by anti-CD3/CD28 (Stemcell, Canada, #10971) and IL-2 (PeproTech). Then CD8^+^ T cells were incubated with CM described above. By gating on FITC, flow cytometry was used to quantify the CFSE dilution 72 h later. The floating cells were collected, tested for apoptosis using Annexin V-APC and 7AAD, and then submitted to flow cytometry to study apoptosis of CD8^+^ T cells. Female C57BL/6 mice were injected with 5×10^6^ MC38-OVA-NC or MC38-OVA-shCD73 cells in the cecum to track the apoptosis of adoptively transferred T cells. The OVA peptide (MBL, Japan, #TS-5001-P) and 50 U/mL IL-2 were used to preactivate OT-1 CD8^+^ T cells for 24 hours. Preactivated OT-1 CD8^+^ T cells were administered intravenously 6 days later. Peritoneal exudate cells (PEC) were collected and stained using 7AAD and Annexin V-APC. The proportion of cells that were gated on the CD8^+^ CD90.1^+^ fraction was used to quantify apoptotic cells.

### Transwell migration and wound healing assays

Prior to conducting the transwell assays, cells were cultured in RPMI 1640 medium without serum to minimize their activity. After 24 h, 50,000 CRC cells were seeded in the upper chamber. In the upper chamber, 300 μl of serum-free RPMI 1640 medium was added. The lower chamber was filled with 700 μl of RPMI 1640 medium containing 10% FBS to act as a chemoattractant for CRC cells. Following a 24-hour incubation in a standard incubator, the upper chamber was removed, and the cells were fixed with 4% paraformaldehyde for 10 minutes, stained with crystal violet for 15 minutes, and then washed with PBS three times. Microscopic images were taken, and cell counts were obtained from five different areas.

For wound healing assays, CD73 OE/KD cells and their respective control cells were seeded in 6-well culture plates following transfection. A linear wound was manually created on the confluent cell monolayer using a standard 20 μl pipette tip. After washing away floating cells with PBS, serum-free medium was added, and the cells were incubated at 37°C. Microscopic images were captured at 0 h and 24 h, and the width of the scratch was measured. Each experiment was performed in triplicate.

### Western blotting (WB) analysis

WB assays were conducted according to conventional methods. Firstly, cells were extracted by RIPA lysis buffer with protease inhibitors and phosphatase inhibitors added (all from Bestbio, China). Then the concentration of protein were examined by BCA kit (Keygen, China). Blots were obtained by using 10% SDS-PAGE gels (EpiZyme, China) and PVDF membrane (Millipore). PBS with 0.1% Tween-20 (PBST) blocking buffer containing 5% milk or 5% Bovine Serum Albumin (BSA) were used to block the blots at room temperature for 1 h. Rabbit antibodies against CD73, ERK, phosphorylated ERK, MEK, phosphorylated MEK, GAPDH, β-actin were purchased from Proteintech Technology (Wuhan, China). The blots were scanned with SuperSignal West Pico chemiluminescent Substrate (Thermo, USA) and Tanon-5200 system (Bio-Tanon, China).

### Immunohistochemistry (IHC) and Immunofluorescence (IF) staining

IHC and IF staining were conducted following protocols in our previous publication 17. Anti-CD73 (#12231-1, Proteintech) and anti-CD8 (ab217344, Abcam) were used as primary antibodies (1:200 dilution). HRP-linked goat anti-rabbit IgG (ZSGB-BIO, China) (1:500 dilution) was used as secondary antibody for the IHC analysis. After performing IHC staining, the tissue microarrays underwent digital scanning using an automated slide scanner, capturing the entire area of each tissue spot for IHC assessment. CD73 expression levels were evaluated semi-quantitatively using the immunoreactive score (IRS). In brief, the IRS (immunoreactive score) was calculated as the product of staining intensity (SI) and the percentage of positive cells (PP). Staining intensity (SI) was categorized as follows: 0 = negative, 1 = weak, 2 = moderate, 3 = strong. Positive cell percentage (PP) was defined as 0 = 0%, 1 = 0-25%, 2 = 25-50%, 3 = 50-75%, 4 = 75-100%. To dichotomize the continuous IRS values into low and high categories, a cutoff point was selected for measurements within the range of 0 to 12 (cut point ≤ 4 versus > 4). Alexa 488/594 conjugated secondary antibody (1:200 dilution) was used for the IF analysis. Cell nucleus were stained with DAPI (#S2110, Solarbio). Olympus confocal fluorescence microscope (Fluoview FV1000) was used for photography. In each sample statistical analysis, three fields were chosen at random.

### Mouse models

All mice utilized in this investigation had a C57BL/6 genetic background., among which the wild-type mice were obtained from the Animal Center of Southern Medical University (Guangzhou, China) and the OT-1 mice (C57BL/6-Tg(TcraTcrb)1100Mjb/Crl) were generously provided by Dr. Wei Yang. Mice were kept in a pathogen-free, temperature-controlled facility with a 12 h light/dark cycle, where they had unrestricted access to food and drink. All experiments were performed in accordance with institutional guidelines approved by the Animal Care and Use Committee.

For tumor growth assays, MC38 control or CD73-silenced cells (both 2 × 10^6^ cells/mouse) were injected subcutaneously into the right dorsal flanks or into the cecum mesentery of 6-week-old wild-type C57BL/6J mice. For subcutaneous tumors, tumor sizes were recorded every 2 or 3 days using vernier calipers. Tumor volume was calculated according to the formula: Volume (mm^3^) = width^2^ (mm^2^) × length (mm)/2. At 4 weeks, mice were executed, and tumors were carefully peeled off and weighed. For colorectal orthotopic tumors, tumor growth was monitored weekly by bioluminescence. At 3 weeks, mice were executed and tumors were carefully peeled off and weighed.

For the adoptive cell transfer assays, MC38 control or CD73-silenced cells (both 2×10^6^ cells/mouse) were injected into the intestine of 6-week-old wild-type C57BL/6J mice via the cecum mesentery. On day 6, pre-activated OT-1 CD8^+^ T cells were injected intravenously into the tumor-bearing mice. Bioluminescence was used to monitor tumor growth weekly.

For the immune checkpoint blockade assays, MC38 cells (2 × 10^6^ cells/mouse) were injected subcutaneously into the right dorsal flanks of 6-week-old wild-type C57BL/6J mice. On days 7, 12 and 17, 10 μg polyclonal rat IgG (clone, LTF-2, BioXcell), anti-PD-1 (clone RMP1-14, Catalog # BP0146, BioXcell) and/or anti-CD73 (clone TY/23, Catalog # BE0209, BioXcell) antibodies per gram of mouse body weight were i.p. into the tumor-bearing mice. Tumor sizes were recorded every 2 or 3 days using vernier calipers. Tumor volume was calculated according to the formula: Volume (mm^3^) = width^2^ (mm^2^) × length (mm)/2. At 3 weeks, mice were executed, and tumors were carefully peeled off and weighed.

### Tumor dissociation and tumor-infiltrating lymphocytes (TIL)/splenocytes isolation

Aforementioned subcutaneous or colorectal tumors were dissected and cut into small pieces. To get single cell suspension, tumors were digested in Hank's Balanced Salt Solution (HBSS) containing 1 mg/mL collagenase type IV, 0.1 mg/mL DNase I, and 1 mg/mL of hyaluronidase (all from Sigma) for 30 min at 37 °C. For isolation of splenocytes, spleens from mice were grinded repeatedly and then washed with PBS. ACK lysis buffer was used to lyse residual red blood cells. Suspension of bulk tumor cells or splenocytes were passed through a 40 μm strainer and washed with PBS.

### Flow cytometry analysis

Antibodies targeting CD3, CD4, CD8, CD45, CD25 and PD-1 (all from Biolegend) or the Annexin V-APC/7AAD dye were added to 100 μL cell suspensions and incubated for 30 min at 4 ℃. For intracellular staining, following a PBS wash, the cells were fixed and permeabilized using BD Cytofix/Cytoperm solution (BD Biosciences), followed by adding GZMB or IFNγ antibody (both from Biolegend) for 30 min at 4 ℃. Then, the cells were resuspended in 200 μL PBS and analyzed on a FACS flow cytometer. The results were analyzed by the FlowJo software.

### Bioinformatics and statistical analyses

Comparison of CD73 expression between colon cancer samples in TCGA and normal colon tissues in GTEx was conducted in the TCGA TARGET GTEx cohort in Xena platform (http://xena.ucsc.edu/). Series matrix files of GSE39582, GSE149206 and GSE178341 were available in GEO databases. Analyses were conducted using GraphPad Prism software for Win8.0 version. All results are presented as means ± SD. Kaplan-Meier method and log-rank test were used for survival analysis. Mann-Whitney U test was used to analyze correlated expression levels of CD73 and effector T cells related genes or immune checkpoints. For significance of difference, (*) means p < 0.05, (**) means p < 0.01, (***) means p < 0.001.

## Results

### CD73 is upregulated in CRC tissues and suggests poor prognosis of CRC patients

**Figure 1:**
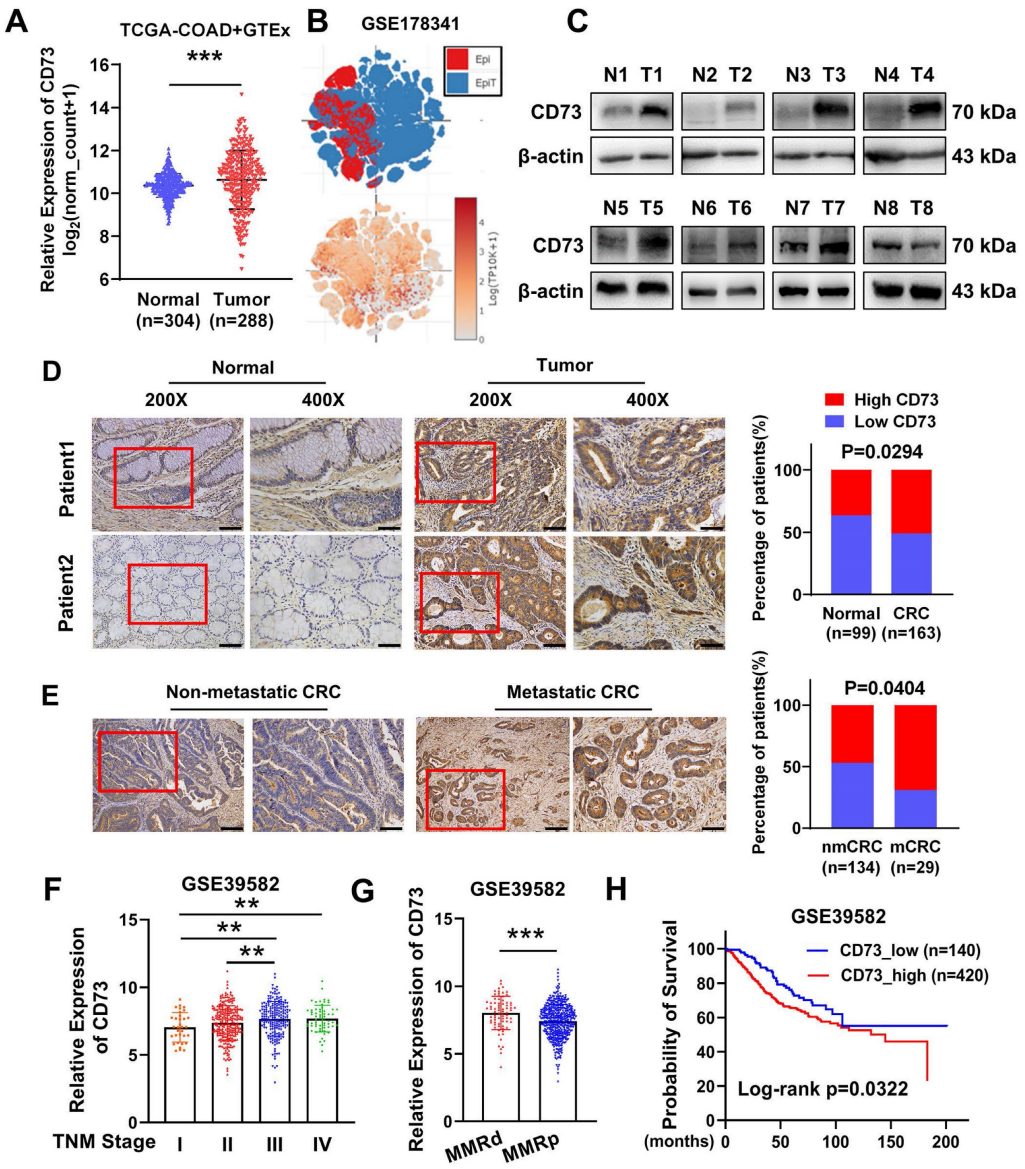
** Overexpression of CD73 is correlated with CRC progression.** (A) CD73 gene expression was compared between TCGA-COAD tumor samples and GTEx normal colon tissues. The analysis was performed online in the TCGA TARGET GTEx cohort in Xena platform (http://xena.ucsc.edu/). (B) tSNE scatter showed distribution and expression level of CD73 in epithelial cells including tumor (EpiT) and non-tumor (Epi) cells in a single cell sequencing dataset. (C) WB assays showed the expression of CD73 in eight pairs of CRC tissues (T) and adjacent non-tumor tissues (N). (D) IHC assays showed the expression of CD73 in CRC tissues (Tumor) and adjacent normal mucosas (Normal) of two representative cases. Bars in the right panel represent the percentage of patients with high or low CD73 expression. The scale bar represents 50 μm. (E) IHC assays showed the expression of CD73 in CRC tissues with or without metastasis. Bars in the right panel represent the percentage of patients with high or low CD73 expression. The scale bar represents 100 μm. (F) The expression of CD73 in stage I-IV CRC samples from the GSE39582 dataset. (G) The expression of CD73 in CRC samples with different statuses of mismatch repair (MMR) in the GSE39582 dataset. (H) The Kaplan-Meier survival curve shows the correlation between CD73 expression and overall survival (OS) in CRC patients from the GSE39582 dataset. Data in A, F-H are presented as means ± SD and analyzed using unpaired two-tailed Mann-Whitney U-test. * p < 0.05, ** p < 0.01, ***p < 0.001.

To explore the role of CD73 in CRC, we firstly compared CD73 expression between colon cancer samples in TCGA and normal colon tissues in GTEx. The findings revealed that CD73 expression of CRC tissues was higher than that of normal tissues (Figure 1A). Accordingly, we inquired CD73 expression in a single cell sequencing 19 and found elevated expression in tumor tissues than adjacent normal epithelial cells (Figure 1B). Then we validated these in our cohort of CRC samples from Nanfang hospital. WB analysis showed an upregulation of CD73 protein in six out of eight CRC tumors, compared to their matched neighboring normal tissues (Figure 1C). IHC results of our cohorts also showed that a higher level of CD73 expression was seen in 50.92% (83/163) of CRC tissues as opposed to 36.36% (36/99) of their matched normal tissues (Figure 1D). More importantly, high CD73 expression was detected in 55.6% (90/162) of the metastatic cases compared with that in 34.5% (20/58) of the non-metastatic cases (Figure 1E). Next, we aimed to explore the correlation of upregulated CD73 expression with clinicopathologic parameters of CRC. Analysis of a dataset of 566 CRC patients showed that CD73 expression in advanced-stage CRC was significantly higher than in early-stage CRC cases (Figure 1F). Mismatch repair (MMR) is an important biomarker for predicting the response to immunotherapy and prognosis. We detected higher CD73 level in MMR-deficient (MMRd) than in MMR-proficient (MMRp) cases (Figure 1G), indicating potential of benefiting from immunotherapies. Kaplan-Meier survival analysis suggested that CD73 expression negatively correlates with CRC patients' overall survival (OS). High CD73 expression indicates worse prognosis (Figure 1H). Collectively, these results suggested CD73 may contribute to progression of CRC.

### CD73 promotes proliferation and migration of CRC cells through the MAPK pathway

**Figure 2:**
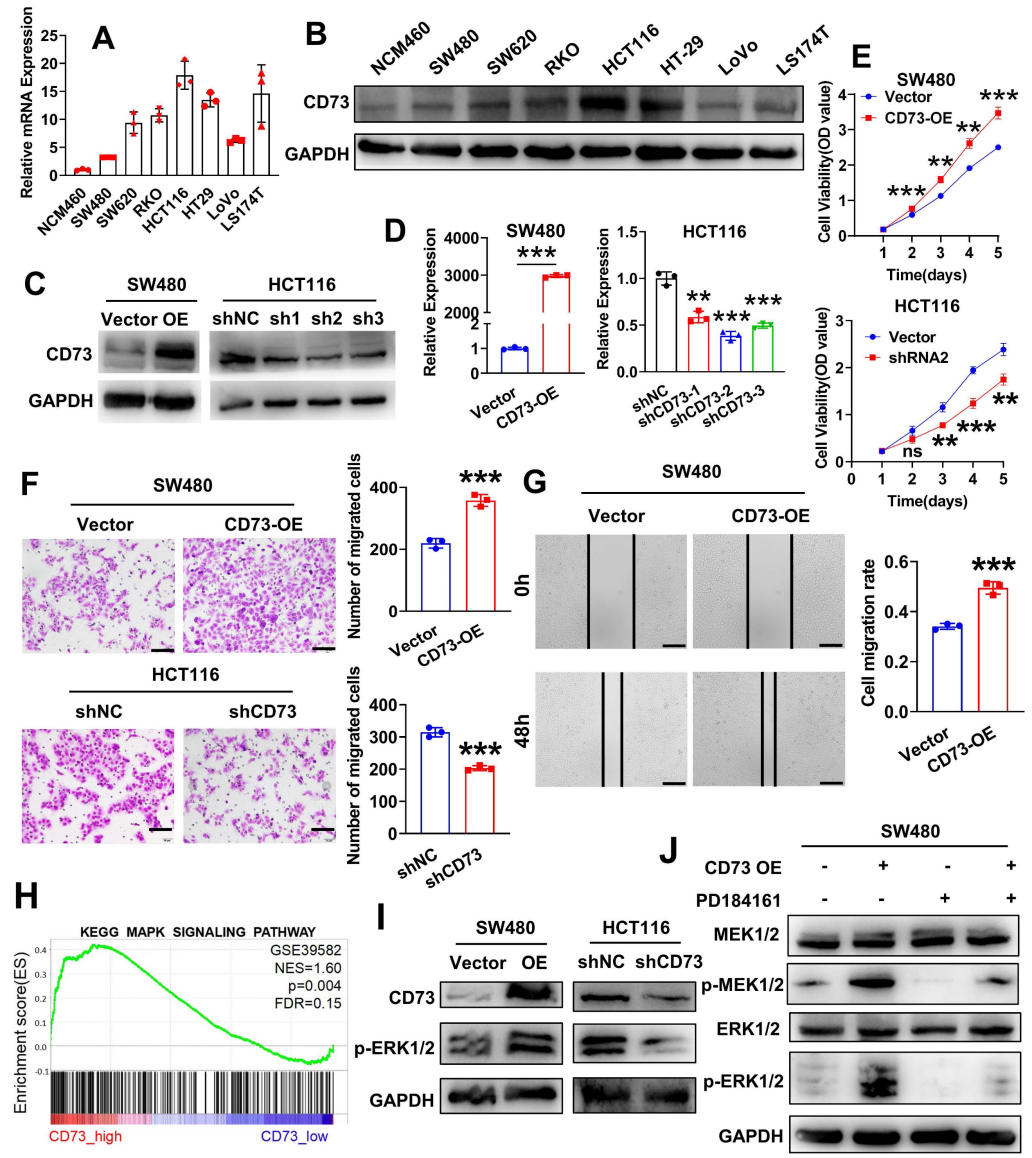
** CD73 promotes proliferation and migration of CRC cells through the MAPK pathway.** (A-B) qRT-PCR(A) and WB(B) assays detected the expression of CD73 in a normal human colon mucosal epithelial cell line NCM460 and CRC cells. (C-D) WB(C) and qRT-PCR(D) assays were performed to verify the successful construction of CD73 overexpression and knockdown CRC cells. (E) CCK-8 assays were performed to determine the effects of CD73 on the proliferation of CRC cells. (F) Representative figures and data of the transwell assays for CD73 overexpression or knockdown CRC cells and their control cells. Bars in the right panel show the number of migrated cells. The scale bar represents 100 μm. (G) Representative figures of the wound-healing assays indicate that CD73 could stimulate migration of SW480 cells. Bars on the right represent the migration rates in two groups (n = 3, p < 0.001). The scale bar represents 100 μm. (H) The result of GSEA on microarray data GSE39582 indicates an enrichment of MAPK signaling pathway in the CD73^high^ samples. (I) WB assays show effects of CD73 expression on the MAPK-ERK pathway in CRC cells. GAPDH was used as a loading control. (J) Rescue experiments via WB assays show effects of CD73 expression on the MAPK-ERK pathway in CRC cells. GAPDH was used as a loading control. The concentration of PD184161 used was 2.5 μM. Data in D-G are presented as means ± SD and analyzed using unpaired two-tailed Student's t-test. * p < 0.05, ** p < 0.01, ***p < 0.001.

To learn more about CD73's functions in the TME, we firstly sought to identify specific cell populations that expresses CD73 in CRC. We analyzed a single-cell sequencing GSE178341 and results showed that CD73 is mainly expressed on epithelial cells (cancerous or non-cancerous), endothelial cells and fibroblasts (Figure S1A). Interestingly, expression of CD73 on immune cells including T cells, myeloid cells and others, is nearly undetectable. We validated these results in a murine CRC model. As shown in Figure S1B-C, flow cytometry analysis of MC38 tumors in mice intestines revealed similar results. Thus, we focus on CD73 on cancer cells. By qRT-PCR and WB, we examined the expression of CD73 in a series of human CRC cell lines (SW480, SW620, RKO, HCT116, HT29, LoVo and LS174T) as well as in normal colon epithelial cells (NCM-460). Similarly, both assays showed that in comparison to NCM460 cells, all CRC cell lines had increased levels of CD73 expression, among which HCT116 cells displayed the highest level and SW480 had a lower level (Figure 2A-B). Next, to investigate effects of CD73 on cancer cells themselves, we established stable CD73-silenced HCT116 and CD73-overexpressed SW480 cells. Western blot and qRT-PCR outcomes demonstrated the successful construction of both CRC cell lines (Figure 2C-D). According to CCK-8 tests, CD73 overexpression dramatically accelerated the proliferation of SW480 cells, while knockdown of CD73 inhibited that in HCT116 cells (Figure 2E). In addition, upregulation of CD73 dramatically increased the migration and motility of SW480 cells *in vitro*, as demonstrated by transwell and wound-healing tests (Figure 2F-G). Oppositely, downregulation of CD73 suppressed migration of HCT116 cells (Figure 2F). Due to CD73 being the primary enzyme responsible for adenosine production, adenosine may mediate these phenotypes in CRC cells. Also, by qRT-PCR, we validated expression of adenosine receptors in our cell lines, among which the A2B receptors are the dominant (Figure S2). Therefore, we treated CRC cells with adenosine and APCP, a specific inhibitor of CD73. We confirmed that APCP effectively inhibited enzymatic activity of CD73 (Figure S3B) without affecting its expression (Figure S3A). The results showed that neither adenosine nor APCP had any impact on the proliferation and migration of CRC cells (Figure S4A-B). Furthermore, WB analysis also demonstrated that adenosine or APCP had no effect on the activation of the MAPK signaling pathway (Figure S5). To elucidate the mechanisms underlying CD73 regulated malignant behaviors of CRC cells, we conducted gene set enrichment analysis (GSEA) on the dataset GSE39582 (n = 566). Analysis results indicated that MAPK signaling is one of the top enriched pathways in CD73 high samples (Figure 2H). Previous clinical data have shown that higher CD73 expression predict for progression-free survival (PFS) benefit from cetuximab in both KRAS wild-type and KRAS-mutant groups of patients 20, implicating expression of CD73 as a potential mechanism of cetuximab action and sensitivity. Therefore, we sought to know the role of CD73 in MAPK signaling. As shown in Figure 2I, overexpression of CD73 upregulated levels of p-ERK1/2, the major effector of MAPK signaling, while knockdown of CD73 downregulated that in CRC cells. Next, PD-184161, an inhibitor of MEK, was applied to inactivate the MAPK signaling pathway in CRC cells. WB analysis confirmed that PD-184161 treatment abolished CD73-induced upregulation of p-MEK and p-ERK (Figure 2J). These results indicated that CD73 on CRC cells might be involved in the MAPK pathway to enhance the aggressive phenotypes of CRC cells *in vitro*.

### CD73 on CRC cells impairs viability and effector functions of CD8^+^ T cells

As an enzyme on cell surface, CD73 sits downstream of the ecto-enzyme CD39 which mediates ATP or ADP degradation to AMP. CD73 then catalyzes the conversion of extracellular AMP to adenosine (ADO).

**Figure 3:**
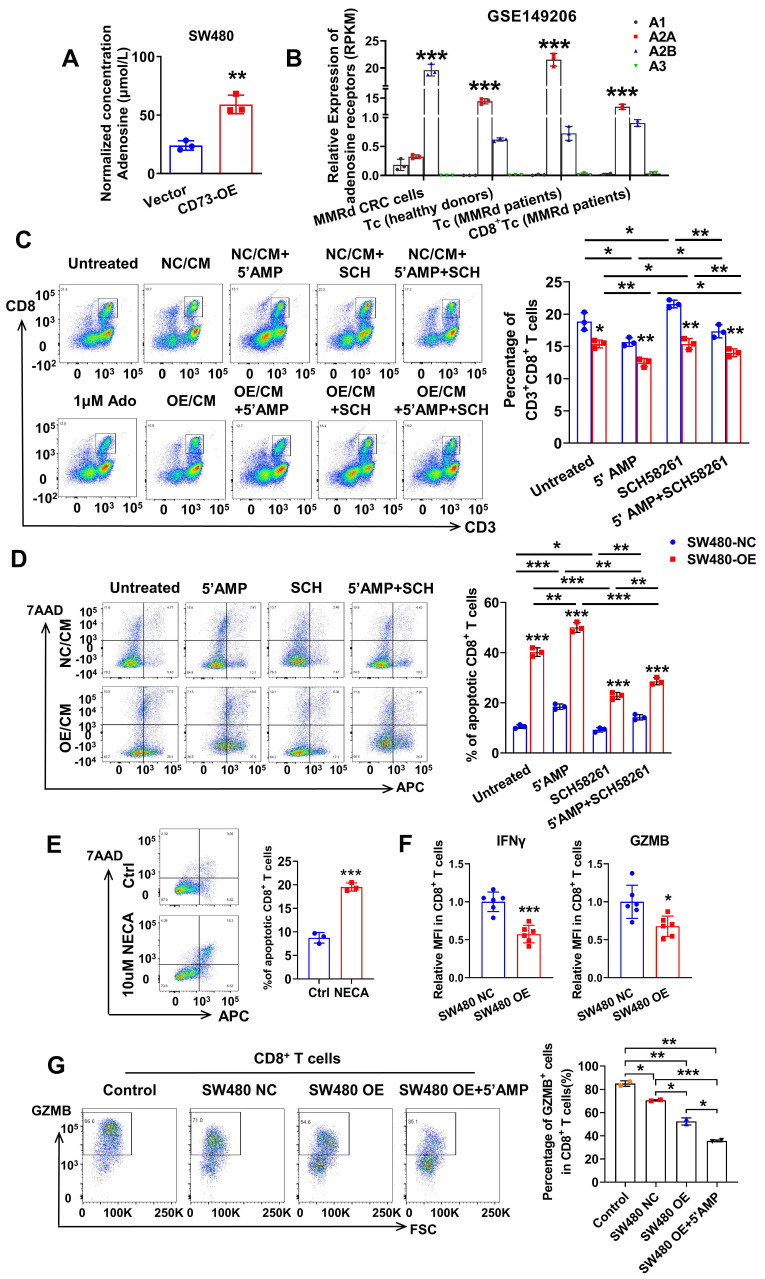
** CD73 on CRC cells impairs viability and effector functions of CD8^+^ T cells.** (A) Concentration of adenosine in the CM of SW480-NC/CD73-OE cells was detected by ELISA. Data was shown as mean±SD from three parallel experiments, n = 3. (B) Expression of four types of adenosine receptors in cancer cells, total T cells, CD8^+^ T cells from MMRd CRC patients and total T cells from healthy donors was analyzed in the GSE149206 dataset. (C) Flow cytometric analyses (left) and quantification (right) of CD8^+^ T cells (CD3**^+^** CD8^+^) in PBMCs treated with CM from SW480-NC/OE cells with or without 5'AMP or SCH58261. Data was shown as mean ± SD, n = 3. (D-E) Flow cytometric analyses of Annexin V-APC and 7AAD staining of apoptotic activated CD8^+^ T cells treated by different CM(D) or NECA(E) for 24 h. Cells for APC^+^/7AAD^-^ and APC^+^/7AAD^+^ were both considered apoptotic. Data was shown as mean ± SD, n = 3. (F) CD8^+^ T cells were preactivated by CD3/CD28 beads plus IL2 for 72 h and then co-cultured with control and CD73 overexpression SW480 cells for 48 h. IFNγ and GZMB on effector CD8^+^ T cells were measured using flow cytometry. Data was shown as mean ± SD, n = 3. (G) CD8^+^ T cells were preactivated by CD3/CD28 beads plus IL2 for 72 h and then co-cultured with control and CD73 overexpression SW480 cells with or without 5'AMP for 48 h. GZMB on effector CD8^+^ T cells were measured using flow cytometry. The concentration of 5'AMP and SCH58261 used was 50 μM and 25 nM, respectively. Data was shown as mean ± SD, n = 3.

Extracellular adenosine functions through interactions with four types of adenosine receptors, including A1, A2A, A2B, A3 receptors. Notably, the four types of adenosine receptors have distinct expression pattens on different tissues and cells. Firstly, we detected the level of ADO in conditioned medium (CM) of CRC cells through enzyme linked immunosorbent assays (ELISA). As expected, concentration of ADO was about 25 μM in CM of SW480-NC cells and three times higher in CM of CD73-overexpression (OE) SW480 cells (Figure 3A). Next, to figure out which adenosine receptor plays a leading role in the TME, we inquired the GSE149206 dataset that includes RNA-sequencing of cancer cells, total T cells, CD8^+^ T cells from MMRd CRC patients and total T cells from healthy donors. Interestingly, the A2AR is the dominant receptor on T cells, while the A2BR is the dominant one on cancer cells (Figure 3B). These findings are consistent with previous reports about roles of A2AR on T cells 21, 22. Considering the dominant role of CD8^+^ T cells in anti-tumor effects, we aimed to study the effects of CD73 on CD8^+^ T cells. We found that comparing to fresh routine medium, CM from SW480 cells decreased proportion of CD8^+^ T cells in PBMCs from healthy donors (Figure 3C). Moreover, the proportion fell further when SW480 cells were treated with AMP. To examine whether ADO functions through A2AR, CM of SW480 cells treated with or without AMP were used to T cell cultures with or without addition of the A2AR inhibitor SCH58261 (Figure 3C). The AMP-induced inhibition of T cell proliferation was markedly reversed by the A2AR blocker SCH58261. Importantly, in all groups, CM of CD73-overexpressed SW480 cells led to a further reduction of CD8^+^ T cells compared to control cells (Figure 3C), indicating tumor CD73-mediated inhibitory effects.

Furthermore, we isolated CD8^+^ T cells exclusively from human whole blood using a specific kit and treated with different types of CM. Similarly, CM from CRC cells inhibited division of CD8^+^ T cells (Figure S6). And as expected, upon knock down of endogenous CD73, proliferation ability of CD8^+^ T cells was restored (Figure S6). Addition of SCH58261 further improved these effects, while increased AMP weakened that (Figure S6). Next, we examined the effects of tumor CD73-mediated adenosine production on viability of CD8^+^ T cells. As shown in Figure 3D, overexpression of CD73 on CRC cells promoted apoptosis of T cells *in vitro*. More T cells suffered apoptosis when incubated with the CM of SW480-OE cells in the presence of AMP, and the addition of the A2AR inhibitor SCH58261 decreased T cell apoptosis, indicating that tumor CD73-derived adenosine enhances T cell apoptosis. Similarly, NECA, the agonist of adenosine receptor, directly induced more apoptosis of activated CD8^+^ T cells (Figure 3E). To assess the function of effector CD8^+^ T cells, flow cytometry were used to evaluate the levels of immune activation markers such as interferon-gamma (IFNγ) and granzyme B (GZMB). PBMC-derived T cells from healthy donors were pre-stimulated and co-cultured with control or CD73 overexpressed CRC cells *in vitro*, followed by flow cytometry analysis. The results showed that the expression of IFNγ and GZMB on CD8^+^ T cells was substantially decreased in the CD73 overexpression group (Figure 3F-G). To exclude that the inhibitory effects on T cells are dependent on the increased number of tumor cells, we treated CRC cells with APCP to inhibit CD73 enzyme activity. Upon the addition of 10 μM APCP to the medium, the ability of CM from CRC cells to induce T-cell apoptosis was significantly reduced (Figure S7). Importantly, there was no significant difference observed between CM from SW480 NC cells and SW480 OE cells, indicating minor impact of increased number of tumor cells (Figure S7). In addition, we investigated whether inhibition of A2AR and A2BR on T cells led to a double effect. Results indicated that PSB1115, a specific antagonist of A2BR, did not ameliorate adenosine-induced CD8^+^ T cell apoptosis, nor did its combination with the A2AR inhibitor result in an enhanced therapeutic effect (Figure S8). Taken together, CD73 on CRC cells impairs viability and effector functions of CD8^+^ T cells.

### Knockdown of tumor CD73 decreases tumor burden and improves anti-tumor T cells infiltration *in vivo*

**Figure 4:**
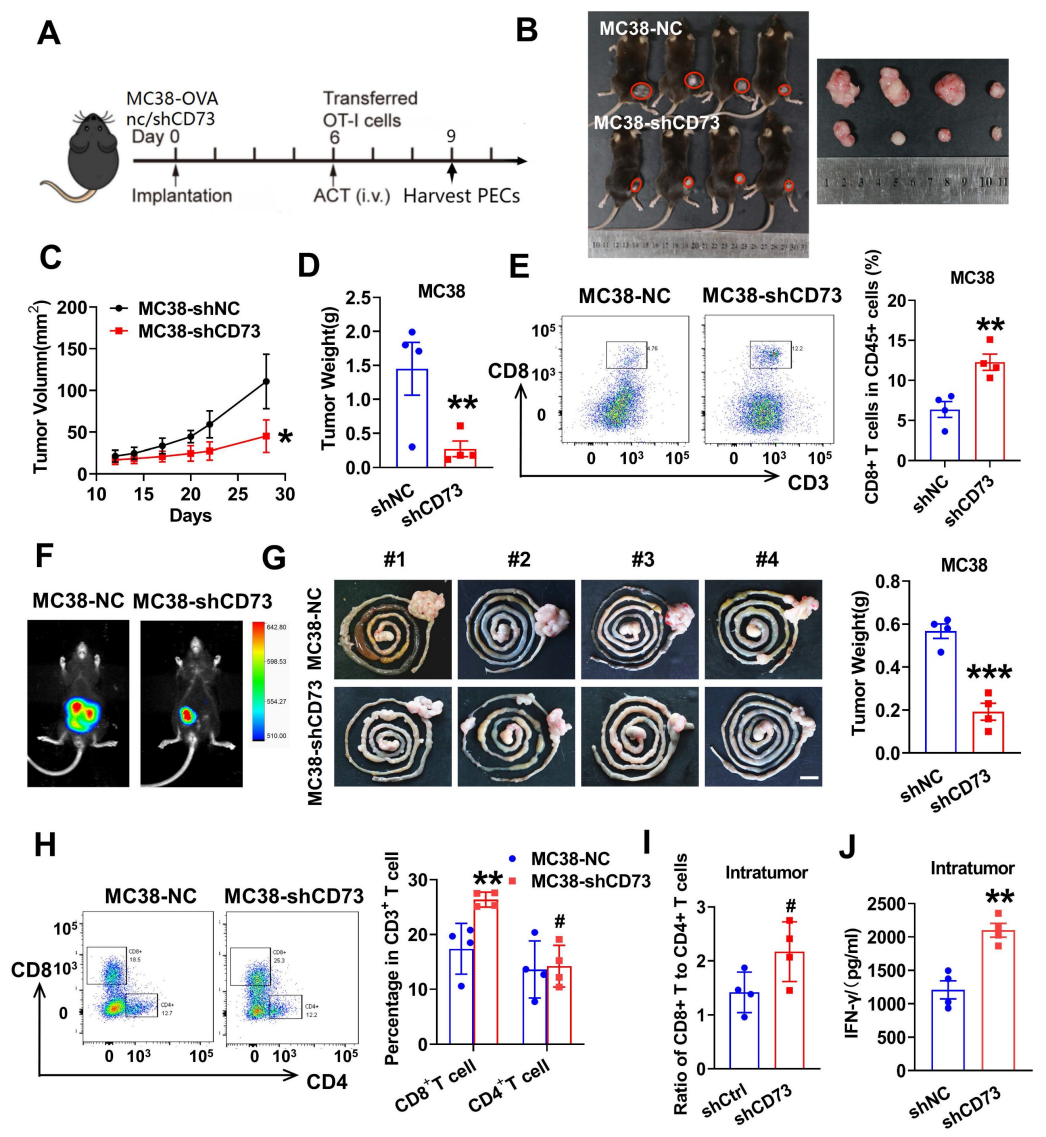
** Knockdown of tumor CD73 decreases tumor burden and improves anti-tumor T cells infiltration *in vivo*.** (A) Treatment regimen of the experiment. Preactivated splenocytes from OT-1 mice were administered by i.v. injection on day 6, n = 4-5. PECs (peritoneal exudates cells) were harvested on day 9. (B) Tumors from LV-control- and LV-shCD73 groups of MC38 subcutaneous tumor model at the end point of experiment. (C-D) Both the volume(C) and weight(D) of subcutaneous tumors were shown. (E) Flow cytometric analyses (left) and quantification (right) of tumor infiltrating CD8^+^ T cells (CD3**^+^** CD8^+^) in subcutaneous tumors from two groups. Data was shown as mean ± SD, n = 4-5. (F) Representative images and quantification of bioluminescence signals in mice injected with MC38-luc cells (control or CD73 knockdown) into intestine (n = 4). (G) Representative gastrointestinal tract samples of two groups are displayed and quantification of tumor weights is placed on the right panel. Sar bar: 1cm. (H) Flow cytometric analyses (left) and quantification (right) of tumor infiltrating CD4^+^ T cells (CD3**^+^** CD4^+^) and CD8^+^ T cells (CD3**^+^** CD8^+^) in orthotopic tumors from two groups. Data was shown as mean ± SD, n = 4-5. (I) Bars show the ratio of tumor infiltrating CD8^+^ T cells to CD4^+^ T cells determined by flow cytometry in two groups. Each spot represents one mouse. (J) Intratumoral IFNγ concentration were determined by ELISA in the orthotopic tumor extracts and then calculated by the total protein concentration. Each spot represents one mouse. * p < 0.05 and ** p < 0.01 by Student's t-tests.

Next, we aimed to explore the impact of CD73 silencing on tumor growth in the immune intact murine models. As shown in Figure 4B-D, growth of subcutaneous tumors in the CD73 knockdown group was inhibited compared to that in the control group. To investigate levels of intratumoral T cell infiltration, single cells from the subcutaneous tumors were separated and subjected to flow cytometric analysis. As a result, two times more CD8^+^ T cells infiltrated the tumors in the CD73 knock down group than the control group (Figure 4E). To better reflect the TME of CRC, we performed orthotopic implantation in mice bowel and monitored tumor growth through bioluminescence. Two weeks after implantation, colorectal tumors formed by control cells were almost three times the size and weight of those formed by CD73-silenced MC38 cells (Figure 4F-G). By analyzing the orthotopic tumors from two groups, increased numbers of CD8^+^ T cells were observed in the CD73-silenced tumors, while CD4^+^ T cells were similar in both groups (Figure 4H). A trend towards an increased ratio of CD8^+^ T cells to CD4^+^ T cells was detected in the CD73 knockdown group compared to the control group (Figure 4I). Furthermore, we explored whether effector functions of intratumor CD8^+^ T cells was affected. Using ELISA assays, we detected increased IFNγ in tumors of the CD73-silenced group compared to that of the control group (Figure 4J). Therefore, CD73 knockdown inhibited tumor growth through modulating CD8^+^ T cells infiltration and production of cytokines in the TME.

### Targeting tumor CD73 improves antigen-specific CD8^+^ T cell responses *in vivo*

**Figure 5:**
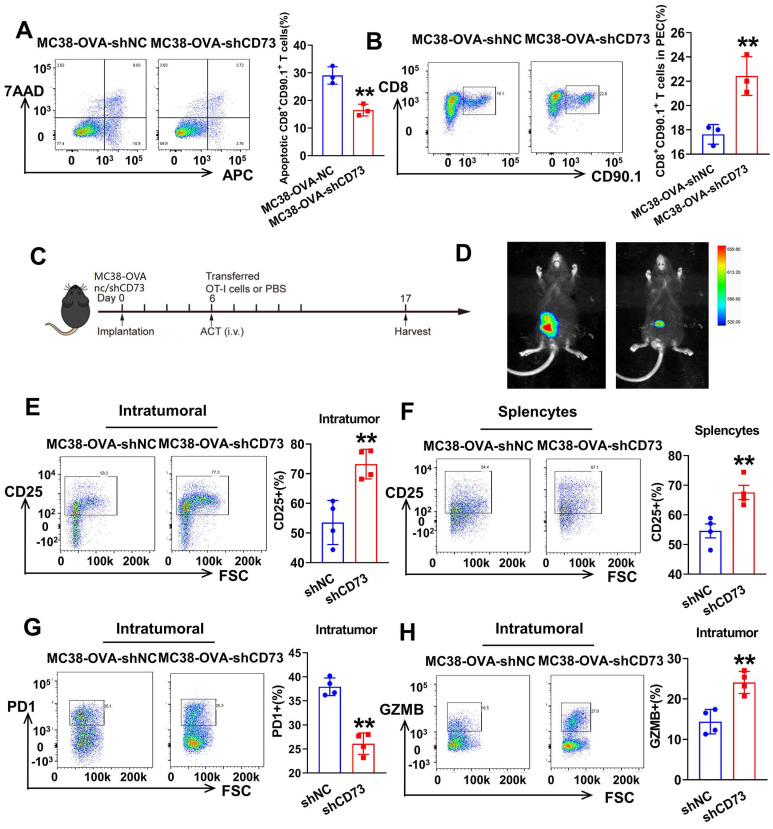
** Targeting tumor CD73 improves antigen-specific T cell responses in OT-1 mice.** (A) Flow cytometric analyses of Annexin V-APC and 7AAD staining of apoptotic activated CD8^+^ T cells in two groups. Cells for APC^+^/7AAD^-^ and APC^+^/7AAD^+^ were both considered apoptotic. Data was shown as mean ± SD, n = 3. (B) Flow cytometric analyses (left) and quantification (right) of tumor infiltrating CD8^+^ T cells (CD8^+^CD90.1^+^) in PECs from two groups. Data was shown as mean ± SD, n = 3. (C) Schematic showing treatment with adoptive transferred OT-1 cells. Tumors were harvested and photographed at the end of experiments (day 17). (D) Representative images and quantification of bioluminescence signals in mice injected with MC38-OVA-luc cells (control or CD73 knockdown) into intestine and treated with transferred T cells (n = 4). (E) Flow cytometric analyses (left) and quantification (right) of tumor infiltrating activated CD8^+^ T cells (CD25^+^) in orthotopic tumors from two groups. (F) Flow cytometric analyses (left) and quantification (right) of activated CD8**^+^** T cells (CD25^+^) in total splenocytes from two groups. (G) Flow cytometric analyses (left) and quantification (right) of tumor infiltrating PD-1^+^ T cells in orthotopic tumors from two groups. (H) Flow cytometric analyses (left) and quantification (right) of effector CD8**^+^** T cells (GZMB^+^) in orthotopic tumors from two groups.

To examine whether tumor CD73 affects response of effector T cells to neoantigen, we established CRC cells expressing OVA (ovalbumin), MC38-OVA-NC and MC38-OVA-shCD73 cells, and conducted orthotopic transplantation in wild-type mice. Next, we activated OT-1 CD8^+^ T cells by OVA *in vitro* and injected i.v. into tumor-bearing mice on day 6. Three days later, peritoneal exudate cells (PECs) were collected and analyzed through flow cytometry (Figure 4A). In the CD73-silenced group, more transferred OT-1 CD8^+^ T cells (CD8^+^CD90.1^+^) were detected (Figure 5B). In the meantime, apoptic transferred OT-1 T cells reduced when CD73 was knocked down, indicating improved viability *in vivo* (Figure 5A). Next, we aimed to study whether targeting tumor CD73 could improve antigen-specific T cell responses using an adoptive cell transfer (ACT) assay (Figure 5C). Two weeks after orthotopic implantation in mice bowels, bioluminescence showed inhibited tumor growth *in vivo* (Figure 5D). On day 17, colorectal tumors were harvested and dissociated into single cells for flow cytometry. CD8^+^ T cells positive for the activation marker CD25 were more in both intratumor and splencytes in the MC38-OVA-shCD73 group compared to the control group (Figure 5E-F), indicating knockdown of CD73 promotes T cell activation. What's more, percentage of PD-1^+^ cells, the major immune checkpoint, fell down and granzyme B (GZMB) expression in CD8^+^ T cells was dramatically enhanced (Figure 5G-H), indicating improved anti-tumor effects.

### Anti-CD73 synergizes with PD-1 blockers to improve tumor immune microenvironment

**Figure 6:**
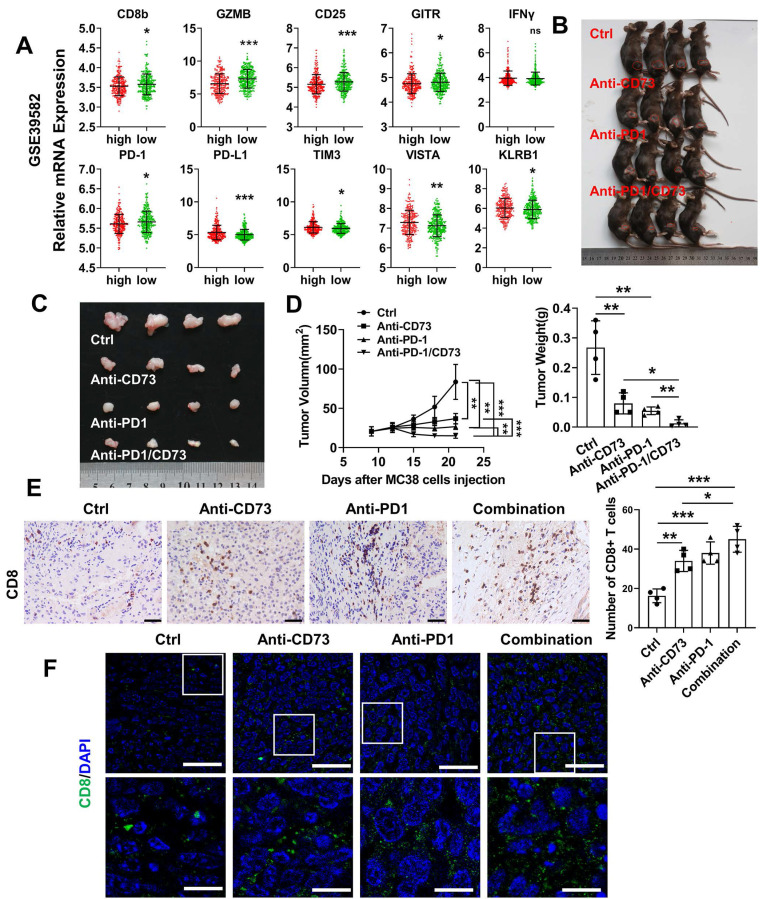
** Anti-CD73 synergizes with PD-1 blockers to improve tumor immune microenvironment.** (A) Relative mRNA expression of markers in patients with high CD73 expression compared with patients with low CD73 expression of the GSE39582 dataset. Means and SDs are shown. Mann-Whitney test was used to calculate statistical significance. * p < 0.05, ** p < 0.01, ***p < 0.001, ns p > 0.05. (B-C) Xenograft subcutaneous tumors formed by MC38 cells followed by treating with different antibodies. (D) The charts show tumor size and the final tumor weight, respectively (n = 4). (E) Representative figures of IHC staining of subcutaneous tumors from the four groups. The scale bar represents 200 μm. Quantification of CD8**^+^** T cells in one field of vision from the IHC staining. (F) Representative figures of IF staining of subcutaneous tumors from the four groups. The scale bar represents 50 μm. The concentration of antibodies used was 10 μg per gram of mouse body weight.

To examine whether CD73 affects activation or exhaustion of T cells in human CRC, we compared CD73 and corresponding markers expression in the GSE39582 dataset. Similar to the observation in the mouse models, T cell markers CD8b, CD25 and the effector molecule GZMB were higher in the CD73-low group. However, level of PD-1 was lower in the CD73-high group, indicating that these CRC patients may not respond well to anti-PD-1 treatments. In addition, important immune checkpoints including PD-L1, TIM3, and VISTA were upregulated in the group with high CD73 expression (Figure 6A). Thus, we asked whether CD73 blockade could improve therapeutic effect of anti-PD1 in CRC. Wild-type mice received subcutaneous injections of MC38 cells. Anti-CD73 and/or anti-PD-1 mAbs were given on days 7, 12, and 17. We discovered that anti-CD73 or anti-PD-1 monotherapy inhibited tumor development (Figure 6B-D). Furthermore, combination of two mAbs elicited strongest anti-tumor effects (Figure 6B-D), suggesting synergistic effects. Decreased tumor growth and weights were shown in Figure 6D. Furthermore, we performed IHC and IF assays to detect infiltration of T-lymphocytes. Monotherapy improved infiltration CD8^+^ T cells compared to the control group. And this effect was further improved by their combination (Figure 6E-F). Taken together, CD73 depletion increased the presence of tumor-infiltrating CD8^+^ T cells, and the combination of CD73 blockade with PD-1 inhibitors resulted in enhanced effects on improving the tumor immune microenvironment compared to single-agent treatments.

## Discussion

In this study, firstly, we demonstrated CD73 was overexpressed in CRC tissues relative to normal colorectal tissues, which is consistent with a previous report 15 and an analysis of TCGA cohorts data 23 that showed colorectal adenocarcinoma is one of the several solid tumors with the highest overall CD73 expression. CD73 has been demonstrated expressed on CD45^+^ immune cells or CD45^-^ non-immune cells across many types of cancer 11. Specially, Piovesan et al. performed quantification of frequency of CD73^+^ cells in CRC samples and found significantly higher CD73 in CD45^-^ cells like stromal and cancer cells than T cells 23. Similarly, Yu et al. demonstrated that CD73 high cells are mostly activated fibroblasts, which are the dominant stromal cells 24. Here, we focused on roles of CD73 on CRC cells.

In a variety of cancer types, including breast cancer 25, 26, hepatocellular carcinoma 27, and gastric malignancies 28, CD73 may facilitate tumor growth. However, it is not fully understood how CD73's non-immunologic function and molecular mechanism contribute to the development of CRC. High intratumoral CD73 levels have been linked to more numerous, bigger colorectal liver metastases (CRLMs) in CRC 29. We looked at CD73's biological activities in CRC cell lines. The proliferation and migration of CRC cell lines were suppressed when CD73 was silenced, according to *in vitro* and *in vivo* investigations. Through GSEA and WB, we discovered that the important MAPK downstream indicators p-MEK and p-ERK were elevated by CD73 overexpression. An essential mechanism through which tumor cells acquire migratory and invasive features is MAPK signaling. Our findings showed that CD73 could enhance CRC cell invasion and motility by triggering MAPK signaling. Notably, cetuximab, a monoclonal anti-EGFR antibody, was shown to have CD73 as a possible predictive marker 20. Our findings thus support relevant preclinical research.

Ecto nucleotidase CD73 and its upstream enzyme CD39 convert ATP to extracellular adenosine, which can be brought on by metabolic changes like hypoxia, pro-inflammatory programs, and activation of carcinogenic pathways 9. More and more evidence points to the role of CD73-adenosine in the regulation of innate and adaptive immune responses as well as immunosuppression 30. Studies have connected increased CD73 expression to poor outcomes in patients with gastric cancer, gallbladder cancer, breast cancer, ovarian cancer, lung cancer and so on 30. In a study of melanoma, CD73 expression was induced in recurrent melanomas or melanoma patients who received anti-PD-1 therapy, suggesting its involvement in acquired resistance mechanism 31. Here, we showed the restoration of CD8^+^ T cell functions following CD73 depletion on cancer cells, which is in line with prior preclinical investigations in which adenosine's suppressive effects on T-cell activity were reduced by targeting CD73 32, 33. Furthermore, we demonstrated the effect of inhibiting CD73 on antigen-specific CD8^+^ T cell responses. Finally, potentiation of T cell responses was demonstrated *in vivo*, where anti-PD-1 plus anti-CD73 treatment of MC38 tumor-bearing mice led to improved anti-cancer immune infiltration in the TME, which was predominantly characterized by increased infiltrating CD8^+^ T cells and decreased tumor burden.

Current clinical trials using ICBs as single-agent therapy have very poor full response rates, which emphasizes the significance of developing novel combinatorial therapies capable of striking an ideal balance between tumor immunity and autoimmune disease. This work emphasizes the advantages of CD73 inhibition for preventing CRC cell malignancies and regaining anti-tumor CD8^+^ T cell activities, hence presenting unique and efficient approaches for upcoming clinical trials of refractory CRCs.

## Supplementary Material

Supplementary figures.
