# Supplementary material for: Dual role of CD73 as a signaling molecule and adenosine-generating enzyme in colorectal cancer progression and immune evasion
Source: Int J Biol Sci. 2024 Jan 1;20(1):137–51. doi: 10.7150/ijbs.87440 (PMC10750288; doi:10.7150/ijbs.87440)
Supplement: Supplementary file 1 — Supplementary figures. [file ijbsv20p0137s1.pdf]

Supplementary figures and figure legends

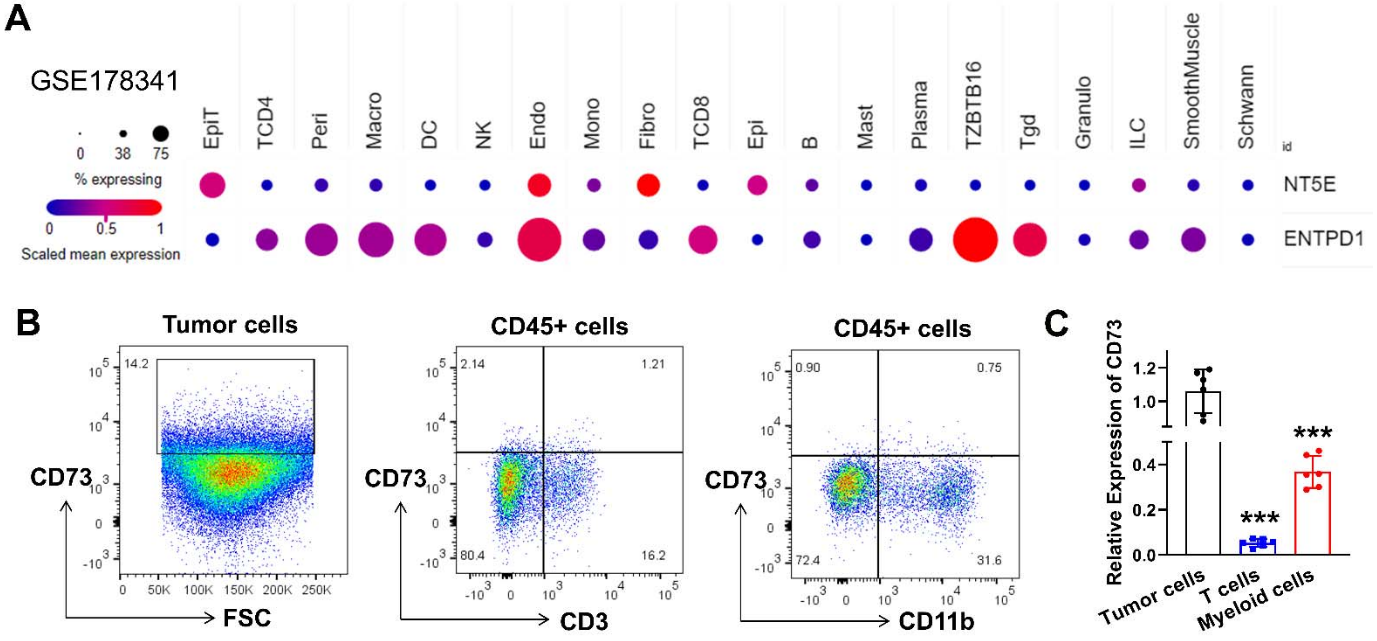

Figure S1. (A) Expression of CD73 (NT5E) and CD39 (ENTPD1) were analyzed in the GSE178341 single-cell sequencing dataset. (https://singlecell.broadinstitute.org/) (B) Flow cytometric analyses (left) and quantification (right) of tumor infiltrating tumor cells (GFP^{+}), T cells (CD3^{+}) and myeloid cells (CD11b^{+}) in orthotopic MC38 tumors. (C) qRT-PCR analysis of CD73 expression in tumor infiltrating tumor cells, T cells and myeloid cells sorted from orthotopic MC38 tumors by flow cytometry.

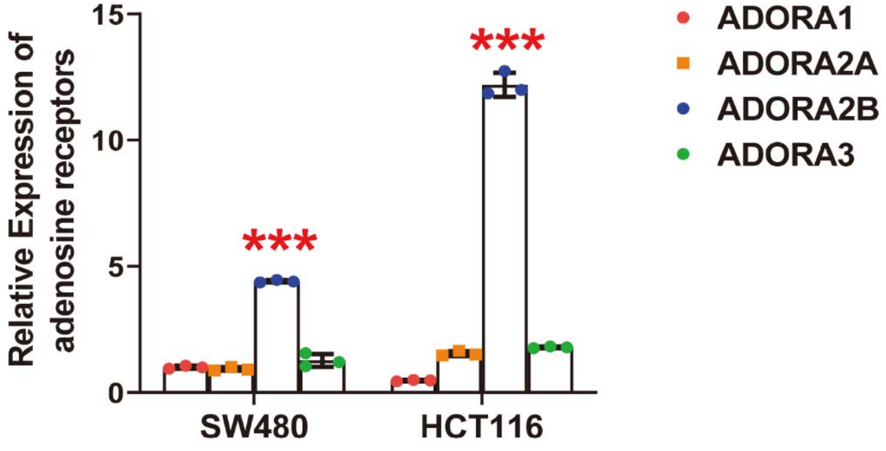

Figure S2. qRT-PCR detected the expression of four adenosine receptors in SW480 and HCT116 cell lines.

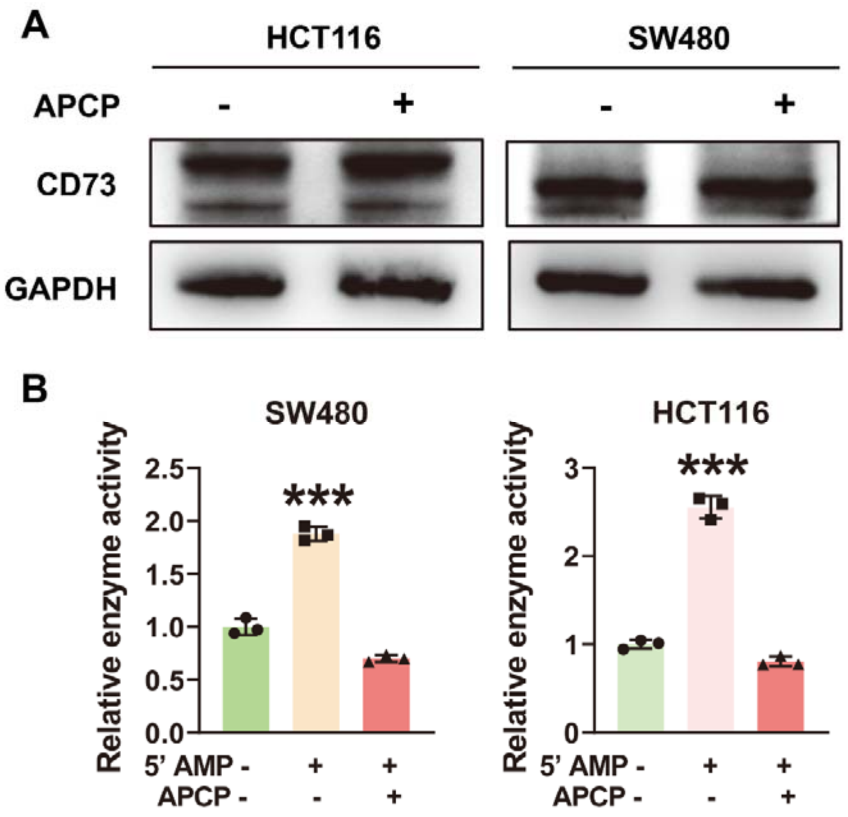

Figure S3. (A) WB assays detected the expression of CD73 upon treatments with 100 µM APCP for 24 h in SW480 and HCT116 cell lines. (B) Malachite green assays were performed to analyze the CD73 enzyme activity upon treatments with 50 µM 5'-AMP and/or 100 µM APCP for 24 h in SW480 and HCT116 cell lines.

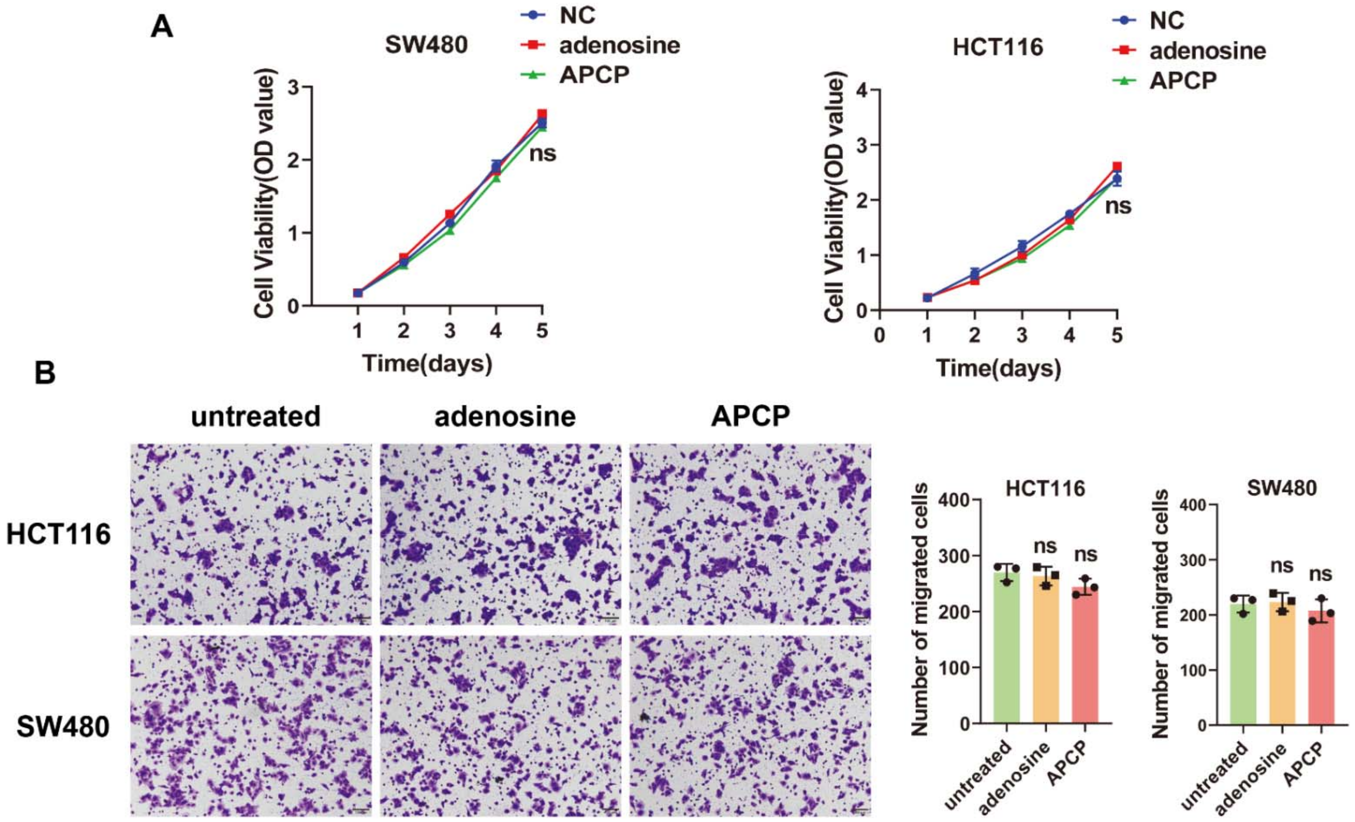

Figure S4. (A) CCK-8 assays were performed to determine the effects of adenosine or APCP on the proliferation of CRC cells. (B) Representative figures (left) of the transwell assays for adenosine or APCP treated CRC cells and their control cells. Bars in the right panel show the number of migrated cells. The scale bar represents 100 μm.

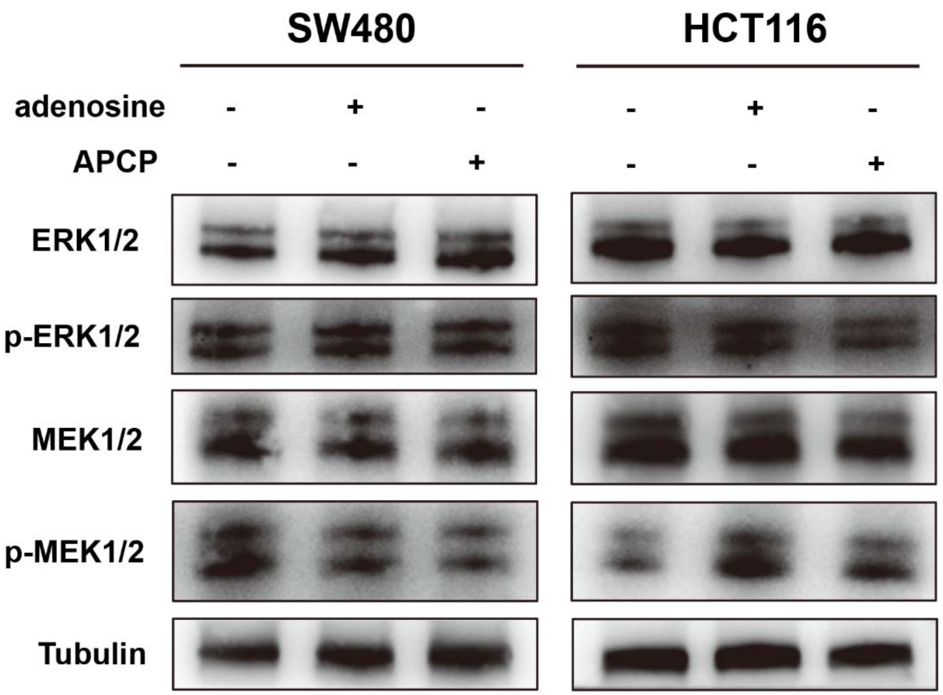

Figure S5. WB assays detected the expression of key downstream effectors of MAPK signaling upon treatments with 100 μM APCP for 24 h in SW480 and HCT116 cell lines.

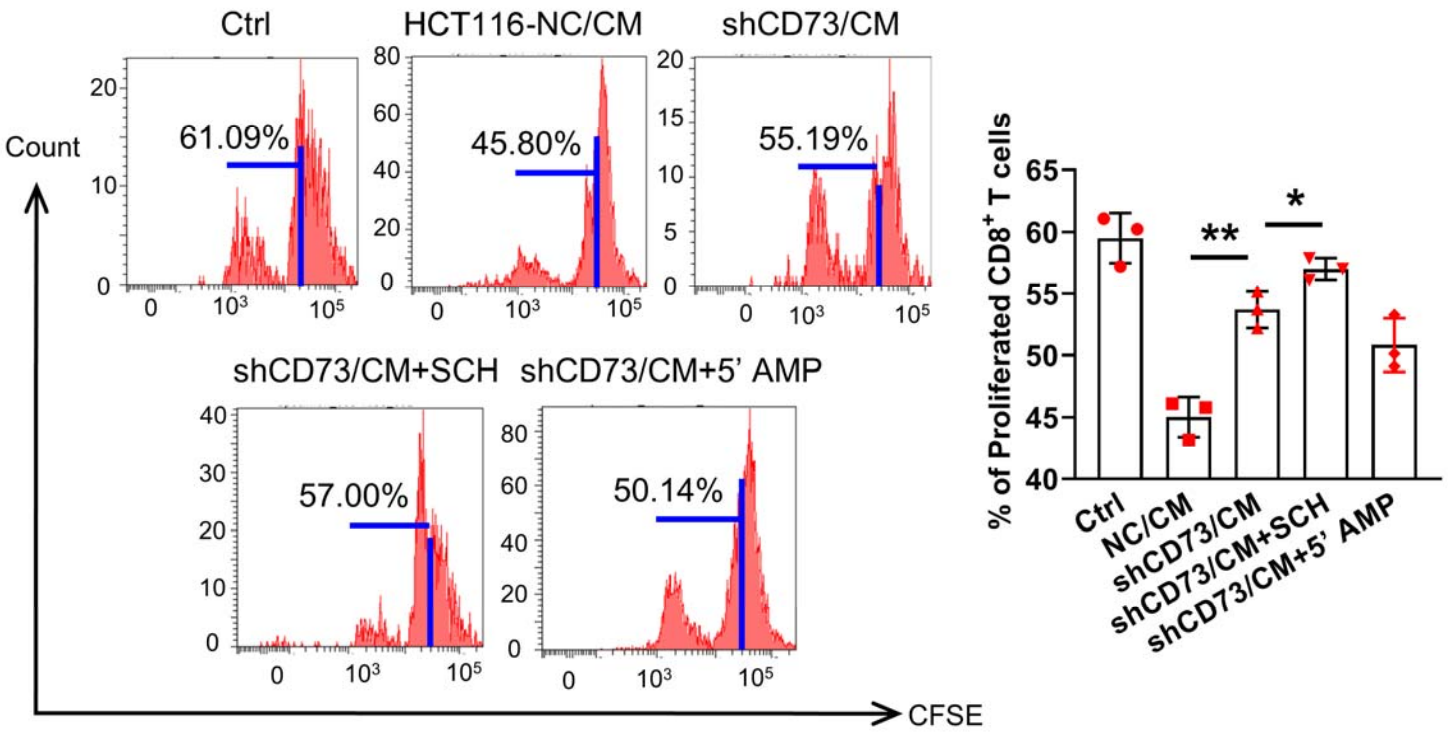

Figure S6. Naive CD8^{+} T cells were extracted from peripheral blood of healthy donors, and then were stained with CFSE and co-cultured with different CM, as described in Fig.3C. Anti-CD3/CD28 antibodies and human IL-2 were added to activate T cells. Quantification of T cells proliferation rate were presented at right. Data was shown as mean±SD from three parallel experiments, n=3.

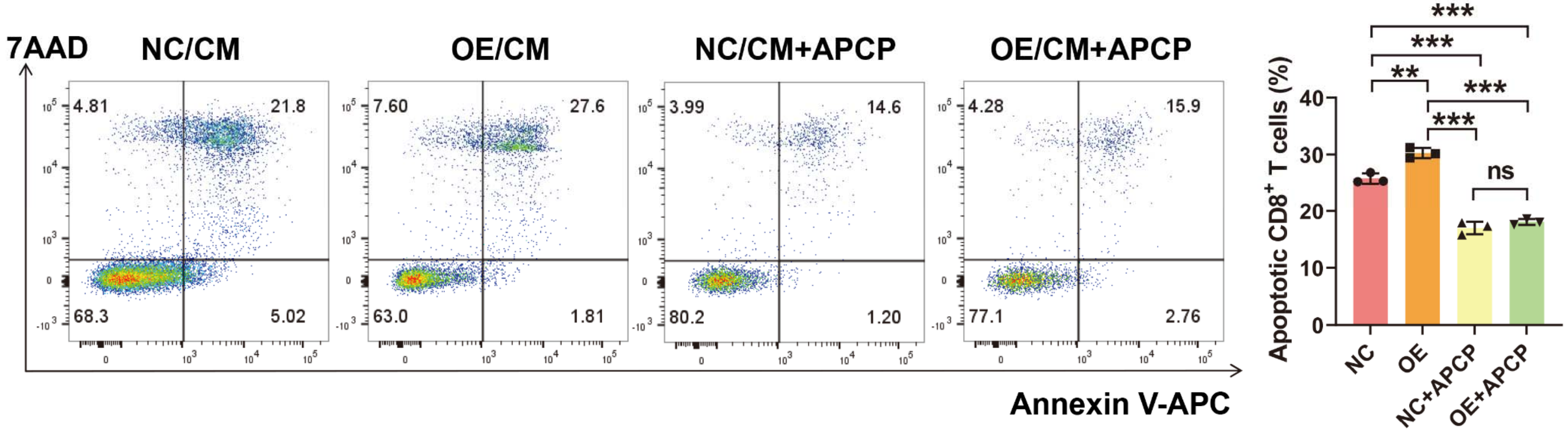

Figure S7. Flow cytometric analyses of Annexin V-APC and 7AAD staining of apoptotic activated CD8$^+$ T cells treated by CM from SW480 NC/OE cells with/without 100 μM APCP for 24 h. Cells for APC$^+$/7AAD$^-$ and APC$^+$/7AAD$^+$ were both considered apoptotic. Data was shown as mean ± SD, n = 3.

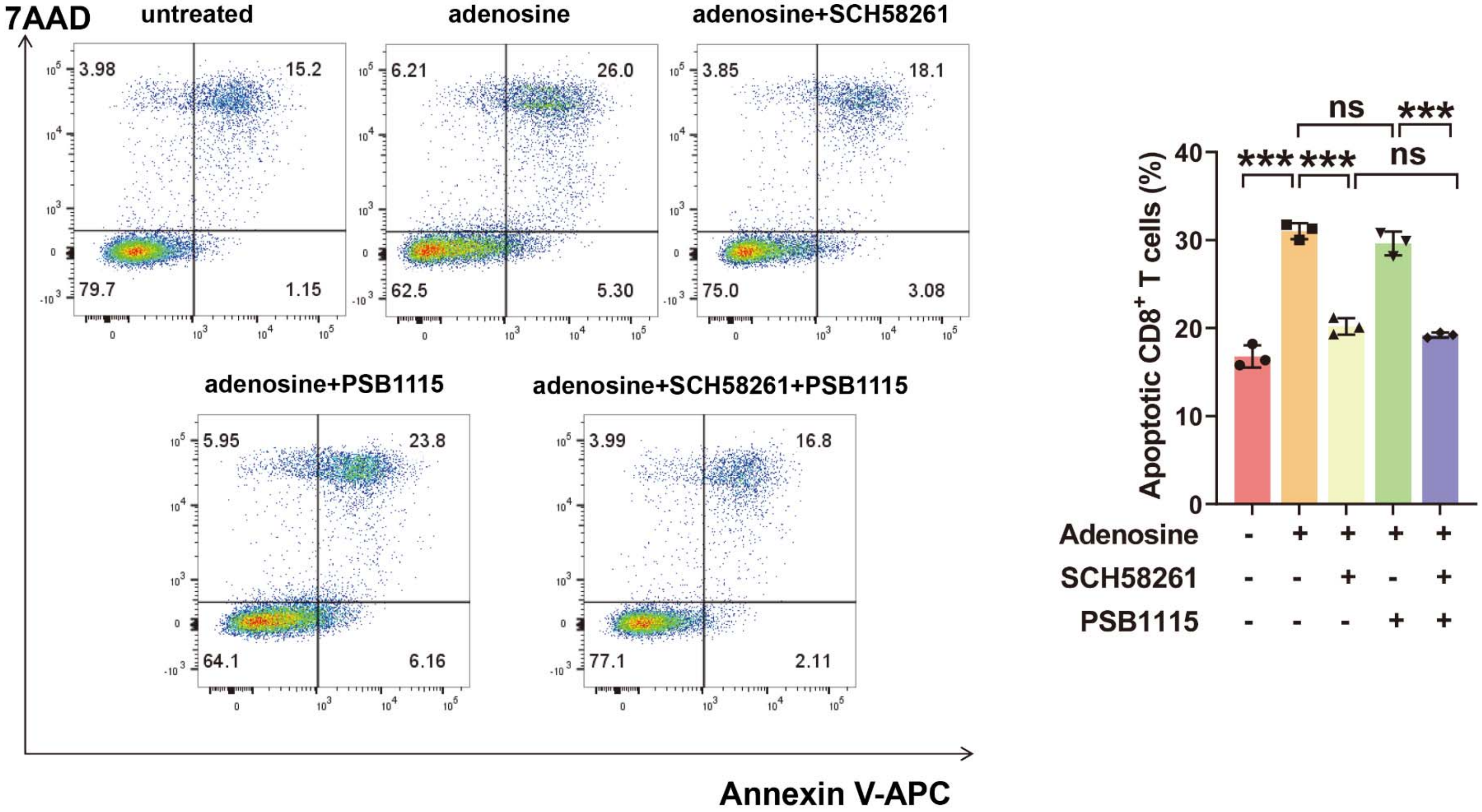

Figure S8. Flow cytometric analyses of Annexin V-APC and 7AAD staining of apoptotic activated $CD8^+$ T cells from different treatments for 24 h. Cells for $APC^+/7AAD^-$ and $APC^+/7AAD^+$ were both considered apoptotic. Data was shown as mean ± SD, n = 3.
